# Transcriptome Analysis Reveals the Variations in Enzyme Production of *Saccharopolyspora rosea* A22 under Different Temperatures

**DOI:** 10.3390/foods13172696

**Published:** 2024-08-26

**Authors:** Congyu Lin, Peiqi Lu, Jingqiu Ma, Zhihui Li, Xiao Han, Zhongwei Ji, Shuangping Liu, Jian Mao

**Affiliations:** 1 School of Food Science and Technology, National Engineering Research Center of Cereal Fermentation and Food Biomanufacturing, Jiangnan University, Wuxi 214122, China; lincongyu@jiangnan.edu.cn (C.L.); 6230111208@stu.jiangnan.edu.cn (P.L.); hanxiao@jiangnan.edu.cn (X.H.); jizhongwei@jiangnan.edu.cn (Z.J.); liushuangping668@126.com (S.L.); 2 Jiangnan University (Shaoxing) Industrial Technology Research Institute, Shaoxing 312000, China; majingqiu1224@163.com; 3 National Engineering Research Center of Huangjiu, Zhejiang Guyuelongshan Shaoxing Wine Co., Ltd., Shaoxing 646000, China; gyls_lzh@163.com

**Keywords:** *Saccharopolyspora rosea*, temperature, saccharifying enzyme, liquefying enzyme, transcriptome analysis

## Abstract

*Saccharopolyspora* is a key microorganism in the fermentation of traditional fermented foods, capable of producing saccharifying and liquefying enzymes at elevated temperatures. However, the specific mechanisms and regulatory pathways governing *Saccharopolyspora*’s response to ambient temperatures are not yet fully understood. In this study, the morphological differences in *Saccharopolyspora rosea* screened from traditional handmade wheat Qu at different temperatures were initially explored. At 37 °C, the mycelium exhibited abundant growth and radiated in a network-like pattern. As the temperature increased, the mycelium aggregated into clusters. At 50 °C, it formed highly aggregated ellipsoidal structures, with the mycelium distributed on the spherical surface. Subsequently, we assessed the biomass, saccharifying enzyme activity and liquefying enzyme activity of *Saccharopolyspora rosea* cultured at 37 °C, 42 °C and 50 °C. Furthermore, transcriptome analysis demonstrated that *Saccharopolyspora rosea* employs mechanisms related to the carbon metabolism, the TCA cycle, glycine, serine and threonine metabolisms, and microbial metabolism in diverse environments to coordinate its responses to changes in environmental temperature, as verified by the expression of typical genes. This study enhances our understanding of the differences in high-temperature enzyme production by *Saccharopolyspora*, and offers valuable guidance for the traditional fermented food industry to drive innovation.

## 1. Introduction

Huangjiu, a traditional Chinese alcoholic beverage, has been cherished by people for millennia [1]. It is characterized by a distinct brewing process, where saccharification and fermentation occur simultaneously, as well as unique flavor profiles, brewing ingredients, types of starter cultures, and microbial interactions. These factors collectively contribute to the distinctive flavor of Huangjiu [2]. The flavor compounds found in Huangjiu are derived from a diverse array of sources, with the microbial metabolism being the primary source responsible for shaping the Huangjiu’s flavor profile [3]. In addition to *Saccharomyces cerevisiae*, other primary microorganisms involved in rice wine fermentation originate from wheat Qu. Wheat Qu harbors a diverse array of microorganisms and metabolites, which serve as a critical driving force for rice wine fermentation and contribute numerous flavor compounds to the final product [4,5]. Research conducted by Xiao et al. [6] and Zhang et al. [7] supports this perspective. Consequently, investigating the composition of the microbial community, the metabolic functions of dominant microorganisms, and the succession patterns within the microbial community in wheat Qu is essential for enhancing rice wine quality and elucidating the mechanisms underlying rice wine flavor formation.

Actinomycetes are prevalent in traditional fermentation processes, particularly in the brewing of rice wine and wine. These microorganisms produce not only antibiotics but also a variety of bioactive hydrolases [8,9,10,11,12]. Wang et al. [13] identified 12 glycoside hydrolases in wheat Qu, including GH13 (α-amylase, EC 3.2.1.1), GH15 (saccharifying enzyme, EC 3.2.1.3), GH5 (cellulase, EC 3.2.1.4), GH16 (endo-1,3(4)-beta-glucanase, EC 3.2.1.6), GH43 (beta-xylosidase, EC 3.2.1.37), and GH47 (lpha-mannosidase, EC 3.2.1.113). These enzymes are associated with the hydrolysis of starch, cellulose, and hemicellulose. GH13 and GH15 are particularly crucial in the fermentation process of rice wine. α-Amylase (GH13), also referred to as a liquefying enzyme, releases a variety of soluble linear and branched glucans from starch, which are subsequently hydrolyzed by α-amylase, isoamylase, or β-amylase to yield short malt oligosaccharides or maltose. This process is vital during the early stages of rice wine fermentation and plays an essential role in initiating the fermentation process. Saccharifying enzyme (GH15) directly releases glucose from starch, glucans, or malt oligosaccharides, providing substrates for subsequent fermentation, which is a key aspect of rice wine production [14]. Therefore, studying liquefying and saccharifying enzymes is of significant importance.

In 1970, *Saccharopolyspora* was first reported by Lacey and Goodfellow [15]. This genus comprises aerobic, Gram-positive, catalase-positive actinomycetes characterized by endo-basi mycelia and aerogenic mycelia. These non-motile organisms propagate in the form of conidia. Sayed et al. [16] confirmed that *Saccharopolyspora* represents a safe biological resource. During the fermentation of wine, Huang et al. [17] isolated *Streptomyces sameri*, *Streptomyces rudyces*, and *Saccharopolyspora* from 50-year-old cellar mud. Wang et al. [18] identified *Saccharopolyspora* as a significant microorganism in the initial phase of Maotai-flavored wine fermentation. Liu et al. [19] reported that *Eurosenia*, *Mirella*, *Streptomyces*, *Saccharopolyspora*, and *Corynebacterium* were prevalent microorganisms in cellar mud from different years. Xu et al. [20] observed that the abundance of *Saccharopolyspora*, *Nocardia*, and *Streptomyces* increased during the first five days of fermentation before beginning to decline. Furthermore, metagenomic sequencing indicated that *Saccharopolyspora* was the predominant microorganism in high-temperature Qu [21,22]. Wang et al. [23,24] also noted that *Saccharopolyspora* was the dominant microorganism in fermentation mash. Other studies similarly identified *Saccharopolyspora* as the dominant bacterium in rice wine fermentation mash, speculating that these organisms primarily originated from wheat Qu [25,26]. Additionally, Gan et al. [27] found that *Saccharopolyspora* can produce enzymes and cellulose-degrading factors that influence the flavor of wine. Thus, *Saccharopolyspora*, as a core microorganism in traditional wine brewing, plays a crucial role, highlighting the importance of further study.

Currently, commercially available rice wine is produced using a starter culture that consists of *Aspergillus flavus* SU-16 cooked wheat Qu and pure *Saccharomyces cerevisiae* HJ wine. However, *Aspergillus flavus* SU-16 exhibits high protease activity, leading to the hydrolysis of raw materials and the production of a significant amount of bitter amino acids [28]. To address this issue, *Saccharopolyspora rosea* A22 was selected from traditional manual wheat Qu as an alternative to *Aspergillus flavus* SU-16 for the production of cooked wheat Qu, which not only reduces the bitter substances but also enhances the flavor profile, particularly the yellow rice wine aroma [29]. During the fermentation process, the cooked wheat Qu rapidly accumulates and heats up to 55 °C, with the resulting biological heat serving a purifying role in the microbial community of the wheat Qu [30,31,32] and minimizing the entry of secondary metabolites from harmful microorganisms into the Huangjiu fermentation system. However, at this temperature, the growth rate of *Saccharopolyspora rosea* A22 slows down, and its ability to produce saccharifying and liquefying enzymes also decreases. Therefore, analyzing the key differentially expressed genes of *Saccharopolyspora rosea* A22 under varying temperature conditions is crucial for understanding the regulatory mechanisms governing its enzyme production. This understanding will aid in enhancing enzyme production capabilities during high-temperature Qu production through modern biotechnological approaches in the future.

## 2. Materials and Methods

### 2.1. Strains and Materials

*Saccharopolyspora rosea* A22 was isolated and deposited in our previous experiments. We conducted 16S rRNA sequence on this isolate, and the resulting sequence is provided in Supplementary Table S1. Actinomycetes medium: KNO_3_ 1.0 g/L, KH_2_PO_4_ 0.5 g/L, MgSO_4_ 0.5 g/L, FeSO_4_ 0.01 g/L, NaCl 0.5 g/L, starch 20.0 g/L, Agar 15.0 g/L. All other reagents were analytically pure.

### 2.2. Cultivation and Morphological Observation of Saccharopolyspora rosea A22

*Saccharopolyspora rosea* A22 was isolated from the glycerol storage tube, streaked onto a solid plate for activation, and incubated at 37 °C for 96 h. Single colonies were then selected and transferred to liquid medium, followed by cultivation in a shaker at 37 °C and 220 rpm for 72 h to generate a primary seed solution. Subsequently, the culture was transferred at a 5% inoculation rate and incubated at 37 °C and 220 rpm for 48 h to obtain a secondary seed medium.

The secondary seed liquid of *Saccharopolyspora rosea* A22 was inoculated into a liquid culture medium and incubated at temperatures of 37 °C, 42 °C and 50 °C with agitation at 220 rpm for 72 h. Subsequently, a suitable volume of bacterial culture was utilized for Gram staining, and the morphological characteristics of the *Saccharopolyspora rosea* A22 were examined using an optical microscope.

### 2.3. Measurement of Biomass

The *Saccharopolyspora rosea* A22 culture liquid was centrifuged at 10,000 rpm and 4 °C for 10 min to separate the cells, after which the supernatant was removed. The resulting cell pellet was then dried at 55 °C for 48 h until a constant weight was achieved. Subsequently, the dried cell pellet was weighed using an electronic balance to determine its dry weight.

### 2.4. Production of Cooked Wheat Qu

Strain culture: The *Saccharopolyspora rosea* A22 seed liquid was prepared according to method 2.2, and the production process of inoculating matured wheat Qu is as follows:

(1) Wheat pre-treatment: High-quality red skin wheat was selected to make wheat Qu and the wheat grains were kept dry. The wheat was screened at the conveyor port of the wheat mill to remove impurities and ensure cleanliness. It was then crushed using a wheat mill, with the roller gap set at 1.7–1.8 mm, resulting in 2 to 3 pieces of wheat per grain to break the wheat peel and expose the starch in the wheat grain.
(2) Moistening material cooking: Approximately 40% of normal temperature pure water (by total mass) was added to moisten the material. Care was taken to add water and wheat in batches to avoid local areas becoming too wet or too dry. The mixture was then placed into a pre-heated boiling steam box and steamed for about 40 min.
(3) Cooling inoculation: The digester was opened, and the material was discharged to reduce the temperature to 45 °C and remove any caking material. All cultures were mixed well and an appropriate volume of cultures (5% of the total mass) was weighed using a clean measuring cylinder and evenly added to the material.
(4) Wheat Qu culture: The mixed wheat Qu was placed in an incubator and the fermentation temperature was set to 37 °C, 42 °C and 50 °C, respectively. The culture was maintained for 7 d, and after fermentation was complete, the wheat Qu was fully dried for use.

### 2.5. Determination of Enzyme Activity in Cooked Wheat Qu

The water content was determined after drying at 105 °C or 24 h, and the enzyme activity of the wheat Qu was assessed using a validated standard method [33,34]. To elaborate, a 5.00 g sample of cooked wheat Qu was immersed in 25 mL of sodium acetate buffer (pH 4.6) containing acetic acid at 40 °C for 1 h. Subsequently, the mixture was centrifuged at 8000 rpm for 10 min. The resulting supernatant was collected as the crude enzyme solution for assessing the activity of saccharifying and liquefying enzymes. The saccharifying enzyme activity and liquefying enzyme activity of wheat Qu were determined according to the method of Liu et al. [35].

The activity of liquefying enzymes is quantified by the amount of soluble starch produced at pH 4.6 and 30 °C. Specifically, one gram of dry wheat Qu yields one gram of soluble starch in 1 h, which constitutes one unit of liquefying enzyme activity (U/g). In a similar manner, the activity of saccharifying enzymes is determined by the quantity of glucose produced under the same pH and temperature conditions. Here, one gram of dry wheat Qu produces one milligram of glucose in 1 h, representing one unit of saccharifying enzyme activity (U/g). 

### 2.6. Transcriptome Sequencing and Analysis

According to the method described in Section 2.2, the secondary seed liquid of *Saccharopolyspora rosea* A22 was inoculated into the liquid culture medium at a volume ratio of 2%. The culture was maintained at temperatures of 37 °C, 42 °C, and 50 °C, with agitation at 180 rpm for a duration of 48 h. Total RNA was extracted using the TRIzol method for quality assessment. Subsequently, rRNA was eliminated, followed by synthesis of the first and second strands of cDNA. The cDNA ends were repaired, splicing was performed, fragments were screened post USER enzyme digestion, PCR amplification was carried out, and sequencing was performed on the Illumina platform following quality inspection. Groups A, B, and C were cultured at temperatures of 37 °C, 42 °C and 50 °C, respectively.

After obtaining the raw data, the FASTP tool was utilized to filter out low-quality reads (defined as reads with a Phred quality score of Q ≤ 20, accounting for more than 50% of the total reads). Subsequently, bioinformatics analysis was performed to investigate differential gene expression, differential gene function annotation and functional enrichment of the reference genome (accession numbers: ASM2564359v1) using the filtered clean reads [36].

### 2.7. Primer Design and Gene Expression Verification

Referring to the culture method in 2.6, the bacteria were incubated at 37 °C, 42 °C and 50 °C, followed by centrifugation, collection, and precipitation. Subsequently, they were washed twice with DEPC water and ground into powder using liquid nitrogen. Total RNA was extracted from the bacteria using TRIzol reagent. Due to the RNA’s instability, it is crucial to maintain a consistently low temperature throughout the extraction process and promptly conduct reverse transcription experiments to generate cDNA. To validate the accuracy of the transcriptome sequencing results, four differentially expressed genes (Amy1, Amy2, ClpX, and DnaB) were selected for fluorescence quantitative PCR. Primers were designed for quantitative analysis of these genes, as outlined in Table 1. RT-qPCR was conducted using GoTaq qPCR Master Mix, and the expression levels were assessed employing the 2^−ΔΔCT^ method.

### 2.8. Statistical Analysis

Three parallel experiments were conducted on each independent sample. All data were statistically analyzed using SPSS 19.0 (IBM Corporation, Armonk, NY, USA). Duncan’s multiple-range test was used to compare data means. *p* < 0.05 was considered statistically significant.

## 3. Results and Discussion

### 3.1. Morphological Comparison of Saccharopolyspora rosea A22 at Different Temperatures

In order to investigate the impact of temperature on the morphology of *Saccharopolyspora rosea* A22 during the growth process in the production of cooked wheat Qu, we selected three temperatures, 37 °C, 42 °C and 50 °C, to simulate the self-heating fermentation stage. Subsequently, we observed the microscopic morphology of *Saccharopolyspora rosea* A22 using an optical microscope (×100).

**Figure 1:**
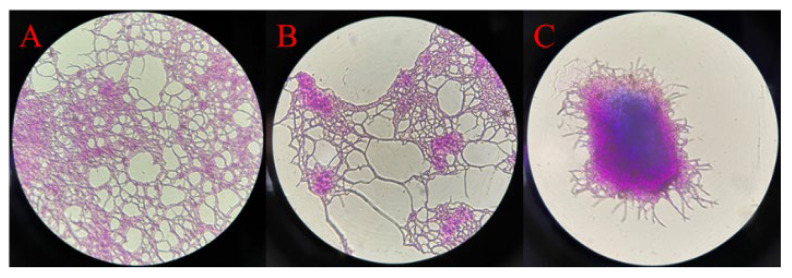
Optical microscope images of *Saccharopolyspora rosea* A22 at different temperatures (×100). (**A**) Optical microscope images at 37 °C; (**B**) optical microscope images at 42 °C; (**C**) optical microscope images at 50 °C.

As depicted in Figure 1A, at a temperature of 37 °C, *Saccharopolyspora rosea* A22 exhibited a proliferation of mycelia visible under a 100-fold optical microscope, demonstrating a distinct polymerization network structure with numerous mycelia radiating outward. At a culture temperature of 42 °C, illustrated in Figure 1B, the network structure of *Saccharopolyspora rosea* A22 began to diminish, causing the mycelia to coalesce into clusters while generating multiple spores connecting to the mycelial ends. Subsequently, at a temperature of 50 °C, as shown in Figure 1C, *Saccharopolyspora rosea* A22 displayed a high degree of polymerization, manifesting an overall ellipsoidal structure with mycelia distributed around the spherical formation.

This morphological variation at different temperatures indicates the phenotypic changes of *Saccharopolyspora rosea* A22 in response to temperature fluctuations. Specifically, 37 °C was identified as the optimal growth temperature for *Saccharopolyspora rosea* A22, facilitating robust growth and proliferation characterized by a complex network structure. Conversely, at 42 °C and 50 °C, *Saccharopolyspora rosea* A22 underwent a reduction in cell surface area through gradual polymerization, thereby minimizing water evaporation and nutrient loss to acclimate to the elevated temperatures. This phenomenon elucidates the progressive transformation of the *Saccharopolyspora rosea* A22 morphology from a network structure to a polymeric spherical configuration with increasing temperature.

### 3.2. Biomass Differences in Saccharopolyspora rosea A22 at Different Temperatures

**Figure 2:**
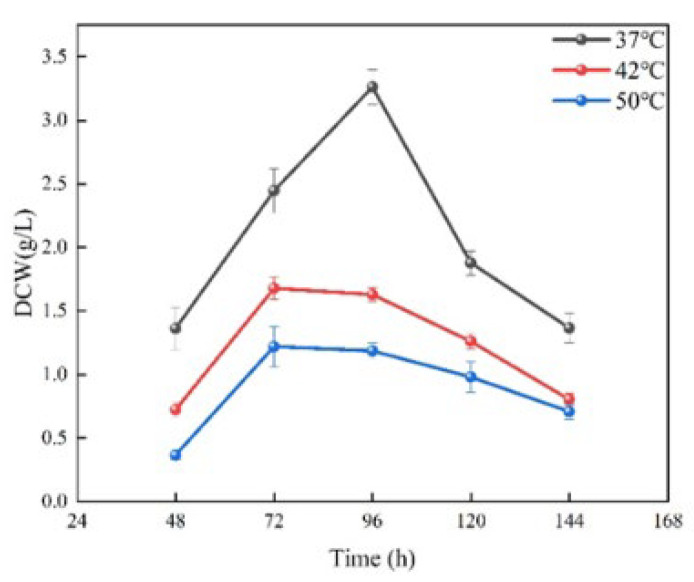
Effect of temperature on the biomass of *Saccharopolyspora rosea* A22 (each group underwent three repeated experiments, with the error bars in the figure representing the standard error).

The biomass differences in *Saccharopolyspora rosea* A22 were assessed at various culture temperatures. As depicted in Figure 2, the biomass trends of *Saccharopolyspora rosea* A22 cultured at 42 °C and 50 °C exhibited a similar pattern, peaking at 72 h, followed by a gradual decline with prolonged culture time. Although the biomass cultivated at 50 °C was slightly lower than that at 42 °C, the difference was not statistically significant. In contrast, at 37 °C, the biomass of *Saccharopolyspora rosea* A22 peaked at 96 h before declining, with the biomass at 37 °C significantly higher than that at 42 °C and 50 °C.

This suggests that a temperature of 37 °C is optimal for the growth of *Saccharopolyspora rosea* A22 compared to other temperatures, resulting in increased biomass accumulation. At 42 °C and 50 °C, biomass accumulation continued to increase, albeit at a slower rate, suggesting that higher temperatures have an inhibitory effect on *Saccharopolyspora rosea* A22 growth. This inhibition may be attributed to cell death, enzyme inactivation, or disruption of other biological processes caused by elevated temperatures, ultimately leading to reduced biomass production.

### 3.3. Comparison of Enzyme Activity in Preparation of Cooked Wheat Qu by Saccharopolyspora rosea A22 at Different Temperatures

Temperature is a critical physical and chemical factor in the fermentation process of wheat Qu, influencing the development of the microbial community. Research indicates that the fermentation process of cooked wheat Qu can be categorized into two stages based on the core temperature: the initial stage (0–48 h) and the subsequent stage (48–168 h) [32]. According to the optimal temperature requirements for various fermentation stages, temperatures of 37 °C, 42 °C and 50 °C were chosen as the culture temperatures to investigate the variations in the activity of saccharifying and liquefying enzymes during the production of cooked wheat Qu using *Saccharopolyspora rosea* A22 at different temperature settings.

**Figure 3:**
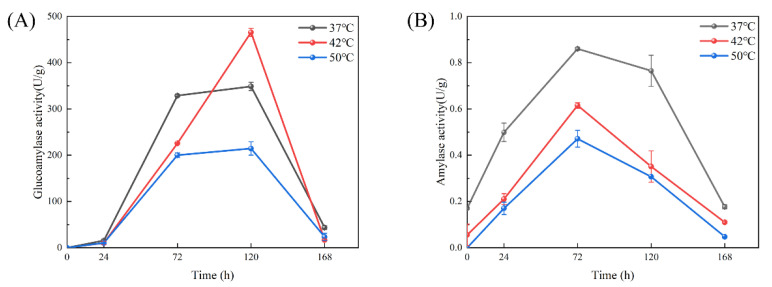
Effect of temperature on the activity of cooked wheat Qu (each group underwent three repeated experiments, with the error bars in the figure representing the standard error). (**A**) Saccharifying enzyme activity; (**B**) liquefying enzyme activity.

The activity of saccharase refers to the ability of saccharase in wheat Qu to break down starch and produce glucose, which is a key index to evaluate the quality of wheat Qu [16]. The activity of mashing enzymes in cooked wheat Qu at different temperatures during the initial stage of fermentation was generally low, as shown in Figure 3A. This may be attributed to the fact that *Saccharopolyspora rosea* A22 was in the rapid growth stage in the first stage of fermentation, rather than the main stage of enzyme production, resulting in a slow increase in the mashing ability of cooked wheat Qu cultured at three temperatures. As the fermentation of cooked wheat Qu progressed to 72 h, the activity of the saccharifying enzymes at different temperatures began to increase rapidly. This was because *Saccharopolyspora rosea* A22 had completed its growth and reproduction process by this time, leading to a significant production of saccharifying enzymes. The activity of the enzymes reached its peak value at 120 h, with values of 368.81 ± 8.83 U/g, 466.51 ± 10.12 U/g and 214.51 ± 14.51 U/g at the three temperature conditions, respectively. To further investigate the differences in enzyme activity at different temperatures, particular attention should be given to the second stage of saccharifying enzyme production. It was observed that from 72 to 120 h, the activity of saccharifying enzymes increased by 20.81 U/g at 37 °C, 239.78 U/g at 42 °C, and 14.31 U/g at 50 °C. Although all three temperatures showed an increasing trend, the mashing power increased more rapidly at 42 °C, indicating that cooked wheat Qu cultured at this temperature exhibited stronger mashing enzyme activity.

The activity of liquefying enzymes reflects the ability of wheat Qu microorganisms and liquefying enzymes to degrade starch [37]. Figure 3B illustrates that, from 0 to 72 h, the activity of liquefying enzymes increased gradually under the three temperature conditions. Upon reaching 72 h of culture time, the activity of the liquefying enzymes peaked. Specifically, the activity of liquefying enzymes under the three temperature conditions was measured at 0.86 ± 0.01 U/g, 0.61 ± 0.01 U/g and 0.47 ± 0.04 U/g, respectively. Subsequently, fermentation continued until 168 h, during which time the activity of liquefying enzymes under the three temperature conditions decreased gradually. It is noteworthy that the trend of liquefying enzyme activity mirrors that of saccharifying enzyme activity. Initially, the activity of liquefying enzymes increased slowly during the rapid growth period of the strain and then escalated rapidly in the subsequent stage. Following the peak, the activity of liquefying enzymes began to decline until the conclusion of the fermentation process. Throughout the fermentation stage, it is evident that the liquefying enzyme activity of cooked wheat Qu under the constant temperature culture condition of 37 °C consistently surpassed that of 42 °C and 50 °C, suggesting that cooked wheat Qu cultured at 37 °C exhibits robust liquefying enzyme activity.

### 3.4. Transcriptome Sequencing Gene Statistics and Sample Relationship

As illustrated in Figure S1, the reference genome contains 5979 genes, with a total of 5900 genes detected in the sequencing process, representing 98.68% of the reference genome. Specifically, 5768 genes were detected in the 37 °C group, accounting for 96.47%, while 5806 genes were detected in the 42 °C group, representing 97.11%. Additionally, 5788 genes were detected in the 50 °C group, making up 96.81% of the total genes. The detailed statistics of the detected gene numbers for each sample can be found in Supplementary Table S2. These results support the validity of the reference genome selection and its suitability for subsequent differential gene expression analysis.

Based on the expression levels of known genes in each sample, principal component analysis and Pearson correlation coefficient were utilized to analyze and assess the repeatability among samples, thereby facilitating the identification and exclusion of outlier samples.

**Figure 4:**
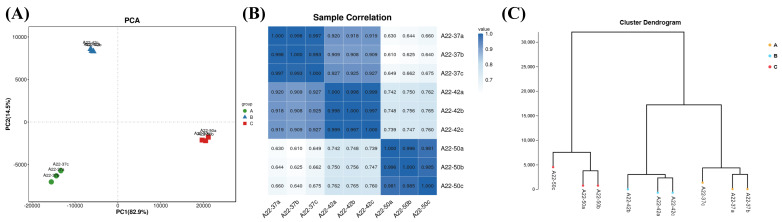
Sample relationships: (**A**) principal component analysis diagram; (**B**) correlation heat map; (**C**) sample clustering diagram.

The R language was utilized to calculate the numerical values of each sample in the first principal component (PC1) and the second principal component (PC2) of the two comprehensive variables. Subsequently, a two-dimensional coordinate diagram, as depicted in Figure 4A, was generated. The analysis revealed minimal differences within each group and no outlier samples were identified. Principal component analysis provides a simple way to visualize the relationship between samples, whereas the correlation coefficient offers a more detailed assessment of the strength of the relationship between samples. To further investigate this, Pearson correlation coefficients were computed between all pairs of samples based on the expression levels of all the genes, and the results are illustrated in Figure 4B. The darker correlation colors among samples within the same group indicate a strong correlation, while lighter colors and weaker correlations are observed between samples from different groups, facilitating the identification of differentially expressed genes. The sample cluster diagram in Figure 4C demonstrates that samples from the 37 °C and 42 °C groups are closely clustered together, whereas samples from the 50 °C group form a separate cluster, suggesting a strong correlation between the 37 °C and 42 °C groups and a notable distinction between the 50 °C group and the others. These findings align with the experimental results about morphology, biomass, and enzyme activity. 

### 3.5. Gene Expression Analysis

**Figure 5:**
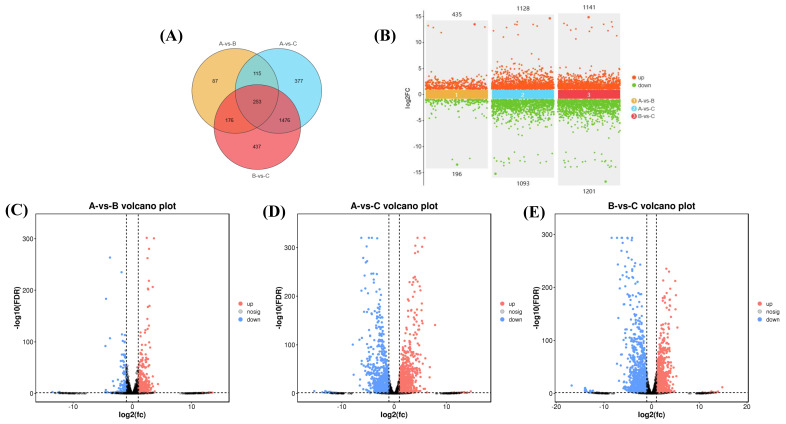
Gene expression analysis. (**A**) Venn diagram of differences; (**B**) multi-group differences: scatter plot; (**C**) 37 °C vs. 42 °C differences: volcano plot; (**D**) 37 °C vs. 50 °C differences: volcano plot; (**E**) 42 °C vs. 50 °C differences: volcano plot.

Figure 5A illustrates the overlap of differentially expressed genes in the various groups. Specifically, there were 631 differentially expressed genes in the comparison between the 37 °C and 42 °C groups, 1093 differentially expressed genes in the comparison between the 37 °C and 50 °C groups, and 1201 differentially expressed genes in the comparison between the 42 °C and 50 °C groups. Additionally, a total of 253 differentially expressed genes were found to be shared among all three groups. Figure 5B presents a statistical summary detailing the upregulation and downregulation of differentially expressed genes within each of the three comparison groups. Figure 5C–E display the volcano maps comparing gene expression between 37 °C and 42 °C, 37 °C and 50 °C, and 42 °C and 50 °C, respectively. It is evident that in the comparison between 37 °C and 42 °C, 435 genes were upregulated and 196 genes were downregulated. For the comparison between 37 °C and 50 °C, 1128 genes were upregulated and 1093 genes were downregulated. Lastly, in the comparison between 42 °C and 50 °C, 1141 genes were upregulated while 1201 genes were downregulated.

### 3.6. Functional Annotation of Differentially Expressed Genes

The coding sequences (CDS) obtained from transcriptome sequencing were compared and analyzed using the Gene Ontology (GO) and Kyoto Encyclopedia of Genes and Genomes (KEGG) databases. Subsequently, the functional information of differentially expressed genes was summarized and analyzed based on the annotations retrieved from these databases.

#### 3.6.1. GO Analysis

**Figure 6:**
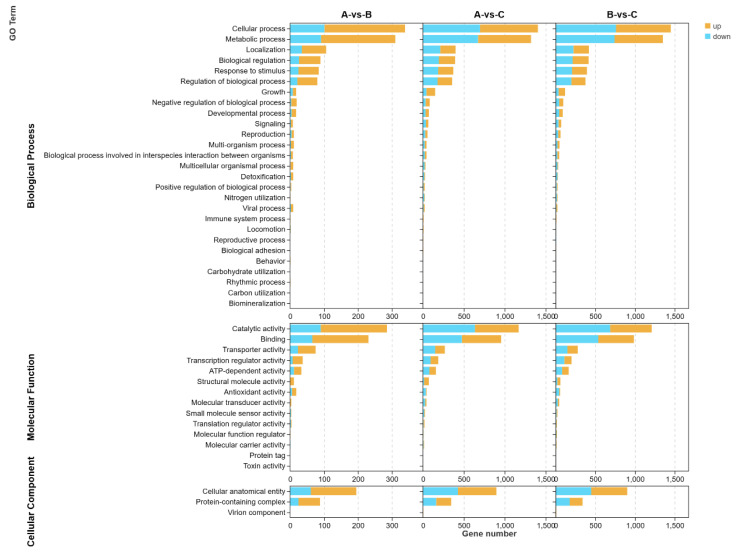
Enrichment of biological processes, molecular function and cellular component in differentially expressed genes.

Through pathway analysis of differentially expressed genes, we can gain insights into metabolic pathways that are significantly altered under specific experimental conditions. Figure 6 illustrates the enrichment analysis of GO functions associated with differentially expressed genes at different temperatures (37 °C for group A, 42 °C for group B, and 50 °C for group C). The comparison of these three groups reveals that the differentially expressed genes primarily involve biological processes such as cellular process, metabolic process, localization, biological regulation, response to stimulus and regulation of biological process. Specifically, when comparing the 37 °C group with the 42 °C group, we observed an upregulation of genes related to cellular process and metabolic process (238 and 219, respectively) which significantly outnumbered the downregulated genes (101 and 91, respectively). On the other hand, the comparison between the 37 °C group and the 50 °C group showed a similar number of upregulated and downregulated genes, while the comparison between the 42 °C group and the 50 °C group indicated a slightly higher number of downregulated genes (759 and 739) compared to upregulated genes (691 and 611) in the categories of localization, biological regulation, response to stimulus and regulation of biological process. Moreover, genes related to growth, negative regulation of biological process, developmental process, signaling, reproduction and other processes also exhibited responses to temperature variations.

The differential genes involved in various molecular functions primarily include catalytic activity, binding, transporter activity, transcription regulator activity, and ATP-dependent activity. When comparing the 37 °C group with the 42 °C group, it was evident that the number of upregulated genes (195, 166, 52, 30 and 22) was significantly higher than the number of downregulated genes (90, 65, 23, 7 and 11). Similarly, in the comparison between the 37 °C group and the 50 °C group, there were more upregulated genes in binding and transcription regulator activities (481 and 95) than downregulated genes (475 and 92). Conversely, for catalytic activity and transporter activity, there were more downregulated genes (635 and 148) than upregulated genes (535 and 119). The number of upregulated genes in ATP-dependent activity was equal to the number of downregulated genes (79). When comparing the 42 °C group to the 50 °C group, the number of upregulated genes (85) in ATP-dependent activity exceeded the number of downregulated genes (78). Furthermore, there were more downregulated genes in structural molecule activity, antioxidant activity and other related genes, indicating a response to temperature differences. 

#### 3.6.2. KEGG Analysis

**Figure 7:**
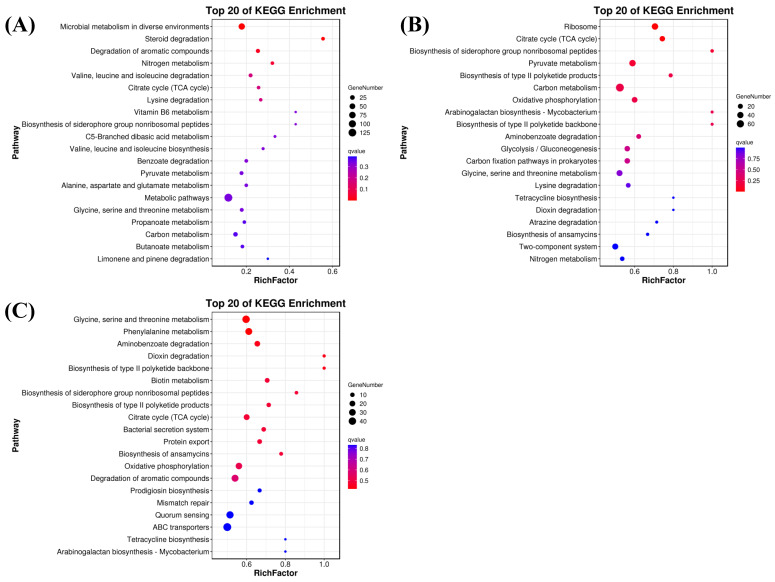
KEGG analysis of differentially expressed genes. (**A**) KEGG analysis of differentially expressed genes at 37 °C vs. 42 °C. (**B**) KEGG analysis of differentially expressed genes at 37 °C vs. 50 °C. (**C**) KEGG analysis of differentially expressed genes at 42 °C vs. 50 °C.

The growth, development, and metabolism of microorganisms require complex coordination of metabolic pathways. Therefore, pathway enrichment analysis of differentially expressed genes can provide a deeper understanding of the functions of these metabolic pathways and their relationships. In the comparison of the 37 °C and 42 °C groups, the top 20 enrichment pathways include the microbial metabolism in diverse environments, metabolic pathways, degradation of aromatic compounds and carbon metabolism, each involving more than 15 differentially expressed genes. Additionally, pyruvate metabolism and glycine, serine and threonine metabolism also include more than 10 differentially expressed genes (Figure 7A). In the comparison of the 37 °C and 50 °C groups, as shown in Figure 7B, the top 20 enrichment pathways include carbon metabolism, pyruvate metabolism, ribosome, glycine, serine and threonine metabolism and the two-component system, all involving more than 35 differentially expressed genes. Furthermore, there are several differentially expressed genes in oxidative phosphorylation, carbon fixation pathways in prokaryotes, TCA cycle, glycolysis/gluconeogenesis and other pathways. In the comparison of the 42 °C and 50 °C groups, as shown in Figure 7C, we found that ABC transporters, glycine, serine and threonine metabolism, quorum sensing, degradation of aromatic compounds and phenylalanine metabolism all contain more than 20 differentially expressed genes. Additionally, oxidative phosphorylation, TCA cycle and other pathways also contained more than 20 differentially expressed genes. Through the above analysis, we observed that microbial metabolism in diverse environments, metabolic pathways, carbon metabolism and ABC transporters were significantly affected by temperature changes. The metabolites produced by these pathways also impact the growth and development of microorganisms and play a crucial role in the differences in enzyme production.

### 3.7. Verification of Key Genes

**Figure 8:**
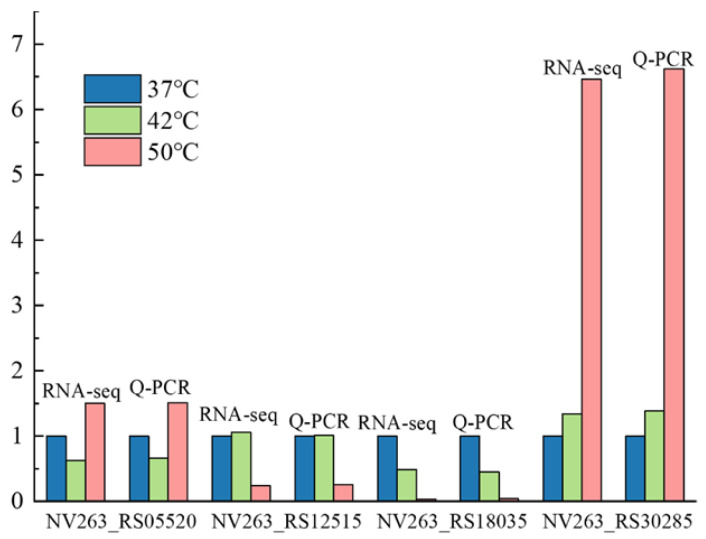
Verification of key genes.

With the temperature of cooked wheat Qu reaching 55 °C during fermentation, a wheat Qu community dominated by *Saccharopolyspora rosea* A22 was established. The biological heat generated during this process played a crucial role in purifying the microbial community in wheat Qu. However, *Saccharopolyspora rosea* A22, a key functional microorganism in wheat Qu used in Huangjiu production, experienced a slowdown in its growth momentum at this temperature. Consequently, its capacity to produce saccharifying and liquefying enzymes also decreased. The growth and fermentation of *Saccharopolyspora rosea* A22 exhibited a declining trend as temperature increased. Consequently, the genes Amy1 (NV263_RS18035), Amy2 (NV263_RS12515), ClpX (NV263_RS05520) and DnaB (NV263_RS30285) were chosen for quantitative analysis. Amy1 and Amy2 are responsible for encoding liquefying enzymes that fall under the category of endo-amylase. These enzymes can break down starch into dextrin and sugar, leading to a rapid decrease in the viscosity of starch paste. On the other hand, ClpX and DnaB are genes that are associated with the growth and development of *Saccharopolyspora rosea* A22. As a member of the Hsp100 family of heat shock proteins, ClpX is highly conserved across various organisms. It plays a crucial role in cell stress tolerance, intracellular protein turnover, DNA replication and gene expression. On the other hand, DnaB is a pivotal protein involved in DNA replication and extension. It functions as a helicase, facilitating the separation of the DNA double helix into single strands in an ATP-dependent manner. DnaB is capable of forming protein–protein interactions with various proteins involved in DNA replication. Specifically, DnaB can interact with the DnaA promoter protein, DnaC loading protein, single-stranded DNA binding protein (SSB), DnaG primase, τ subunit of DNA polymerase and replication termination protein. These interactions, spanning from initiation to termination, highlight the crucial role of DnaB helicases in the process of DNA replication. The results of fluorescence quantitative PCR analysis for differential gene expression are presented in Figure 8. The findings from the fluorescence quantitative PCR analysis of the four selected differential genes align with the results obtained from transcriptome sequencing, demonstrating a consistent pattern of upregulation and downregulation in gene expression. This concordance indicates the reliability of the transcriptome sequencing results. 

With the continuous improvement in living standards, modern mechanized Huangjiu production in the original market is no longer able to meet consumer demand. The concept of preserving traditional brewing methods while innovating in food integrity has gained traction, leading to a resurgence in the popularity of traditional handmade Huangjiu. Therefore, it is crucial to research the core microorganisms involved in this process [29]. Several studies have demonstrated the presence of *Saccharopolyspora* in cellar mud, high-temperature Qu, and fermented mash [38]. *Saccharopolyspora* is classified as an aerobic actinomycete, with environmental temperature playing a crucial role in its growth and metabolism [16]. Typically, the optimal growth temperature for bacteria is 37 °C. In the production of traditional fermented foods, *Saccharopolyspora* is commonly utilized in the preparation of Qu, where the heat generated during fermentation can elevate the core temperature to 52–55 °C. This elevated temperature serves to eliminate other microorganisms in the environment that may hinder the fermentation process, thereby ensuring product quality. Despite the longstanding use of this traditional brewing method spanning centuries, the heat resistance mechanism of *Saccharopolyspora* remains inadequately explored [29]. In this study, a strain of *Saccharopolyspora rosea* A22 was cultured at various temperatures. As depicted in Figure 1, it is evident that *Saccharopolyspora rosea* A22 exhibited robust growth at 37 °C. Following incubation at elevated temperatures, the mycelia underwent contraction and formed dense polymerization, with minimal mycelia dispersed on the periphery. These morphological alterations provide concrete evidence that temperature significantly impacts *Saccharopolyspora rosea* A22, thus warranting further investigation. As depicted in Figure 2, a temperature of 37 °C is identified as the optimal temperature for cell accumulation, resulting in significantly higher biomass compared to *Saccharopolyspora rosea* A22 cultured at 42 °C and 50 °C. As the temperature rises, the growth of the cells is impeded. Nevertheless, at 50 °C, A22 continues to grow albeit at a slower rate with reduced cell accumulation, indicating its robust tolerance to high temperatures. As illustrated in Figure 3, the study examined the activity of saccharifying and liquefying enzymes of *Saccharopolyspora rosea* A22, revealing a significant influence of ambient temperature on both enzyme activities. Specifically, at 120 h, the saccharifying enzyme activity peaked at all ambient temperatures. Subsequently, as the system temperature approached 42 °C, there was a notable increase in the production of saccharifying enzymes. Conversely, at 72 h, the liquefying enzyme activity peaked across all ambient temperatures, with the activity at 37 °C surpassing that at 42 °C and 50 °C. This suggests that liquefying enzyme production occurred predominantly before a sharp temperature increase during the early stages of fermentation. While *Saccharopolyspora* can thrive within a broad temperature range, fluctuations in ambient temperature significantly impact biomass synthesis and secondary metabolite production, leading to observable variations. Notably, the temperature range of 37–42 °C appears to be the most conducive for the growth of *Saccharopolyspora rosea* A22. In general, as ambient temperature gradually increases, microorganisms initiate homeostasis protection mechanisms to strengthen the metabolic pathways in response to temperature changes in order to prevent damage and maintain basic life functions. This may lead to a weakening of certain enzyme production pathways. For example, in this experiment, a significant reduction in enzyme activity was observed, along with the secretion of “sticky substances” causing mycelia to polymerize and form clumps [39]. This phenomenon is also reflected in the transcriptome through differential changes in genes related to microbial metabolism in various environments.

Ambient temperature has a significant impact on the growth, differentiation, and secondary metabolism of microbial cells [40,41]. In order to elucidate the genetic basis of variations in morphology, biomass, and enzyme activity under different temperature conditions, transcriptomic analysis was conducted at 37 °C, 42 °C and 50 °C. The study revealed that secondary metabolite synthesis, cellular respiration and energy production were the predominant biological processes at lower temperatures, especially at 42 °C and 50 °C, when comparing biological processes between 37 °C and 42 °C. The enrichment analysis of transmembrane transporter activity identified over 260 genes (Figure 6). This study elucidates the reasons behind the higher activity of saccharifying enzymes and liquefying enzymes at temperatures between 37 °C and 42 °C compared to 50 °C. By comparing the optimal growth temperature of 37 °C with other temperature levels, this research identifies the key biological processes involved in temperature adaptation and response to the external environment. Notably, when comparing 37 °C to 50 °C, processes such as antibiotic biosynthesis, monosaccharide biosynthesis, cellular homeostasis, transmembrane transport and various other processes are enriched. These processes play a crucial role in mitigating the impact of extreme environmental conditions on microorganisms. The terpenoid biosynthetic process and carotenoid biosynthetic process contribute to enhancing the cell’s environmental tolerance. These processes were observed in the control groups exposed to temperatures of 42 °C and 50 °C, indicating a potential adaptive mechanism of *Saccharopolyspora* to varying environmental temperatures. Through these analyses, we observed a significant decrease in the expression levels of the key genes Amy1 and Amy2, which encode liquefying enzymes, as temperature increased. This decline in gene expression accounts for the reduction in liquefying enzyme activity observed in Figure 3B. Additionally, the expression levels of the growth- and development-related genes ClpX and DnaB also exhibited a decreasing trend with rising temperatures. These findings indicate that elevated temperatures adversely affect the growth of *Saccharopolyspora rosea* A22. The trends identified in the transcriptome analysis corroborate the results obtained from the verification experiment, thereby supporting the findings presented in Figure 2. Akira et al. investigated the DnaK gene within the same family and discovered its function in heat sensing, which aligns with our research findings. This type of gene facilitates biological adjustments in response to rising temperatures [42]. Additionally, a schematic diagram illustrating the microbial metabolism of *Saccharopolyspora rosea* A22 at different temperatures is provided in Supplementary Figure S2. This diagram offers valuable technical support for future research endeavors to delve deeper into this topic.

## 4. Conclusions

This study initially investigated the morphological differences in *Saccharopolyspora rosea* cultivated at varying temperatures and found that as the temperature increased, the phenotype of *Saccharopolyspora rosea* A22 exhibited significant changes. These alterations are indicative of the strain’s adaptive responses to environmental stressors. Subsequently, the effects of different temperatures on biomass accumulation and enzyme production activity were examined. Results indicate that *Saccharopolyspora rosea* A22 can thrive across a broad temperature range; however, its enzyme production activity is markedly influenced by temperature variations. These findings align with established production practices. The operational protocols for workers in the facility include laying the wheat Qu flat at 37 °C, stacking it at 42 °C, and turning it at 50 °C. Furthermore, transcriptome analysis revealed that *Saccharopolyspora rosea* A22 can modulate the carbon metabolism, TCA cycle, glycine, serine, and threonine metabolism, as well as the microbial metabolism in diverse environments to adapt to temperature fluctuations. The quantitative analysis of key genes corroborated this perspective.

## Figures and Tables

**Table 1:** Primers used in this study.

Gene ID	Name	Sequence (5′ to 3′)
Amy1F(NV263_RS18035)	Amy1F	CGCTGACCTGAAAACCGAA
	Amy1R	TGACCGTTGAAGGTGTAGC
Amy2F(NV263_RS12515)	Amy2F	ATGGCTTCCTCGCTGGTCA
	Amy2R	GTTGCTGCCCTCCTTGTCC
ClpXF(NV263_RS05520)	ClpXF	GATGAGCCCGTACTTGATC
	ClpXR	CGGCTGCGGCAAGACCTAC
DnaB(NV263_RS30285)	DnaBF	GCTACCACGGCTCCGAGGG
	DnaBR	CGGGAGAACTCGGAGACTT

## Data Availability

The original contributions presented in the study are included in the article and Supplementary Materials, further inquiries can be directed to the corresponding author.

## Supplementary Materials

The following supporting information can be downloaded at: https://www.mdpi.com/article/10.3390/foods13172696/s1, Table S1: The 16S rRNA sequence of *Saccharopolyspora rosea* A22; Table S2: The number of genes detected in each sample was counted; Figure S1: Statistical map of the number of genes identified; Figure S2: Microbial metabolism in diverse temperatures.
