# Supplementary material for: Transcriptome Analysis Reveals the Variations in Enzyme Production of Saccharopolyspora rosea A22 under Different Temperatures
Source: Foods. 2024 Aug 26;13(17):2696. doi: 10.3390/foods13172696 (PMC11394526; doi:10.3390/foods13172696)
Supplement: Supplementary file 1 [file foods-13-02696-s001.zip › foods-3141541-supplementary.pdf]

Supplementary

Table S1. The 16S rRNA sequence of *Saccharopolyspora rosea* A22

Microorganism	Sequence
Saccharopolyspora rosea A22	CCCCCCGGAAACCGGTTGGGCCATGGGCTTCGGGTGTTACCGACTTTCATGACGTGACG<br>GGCGGTGTGTACAAGGCCCGGGAACGTATTCACCGCAGCACTGCTGATCTGCGATTACT<br>AGCGACTCCGACTTCACGGGGTCGAGTTGCAGACCCCGATCCGAACTGAGACCGGCTTT<br>AAGGGATTCGCTCCACCTCACGGTATCGCCACCCTCTGTACCAGCCATTGTAGCATGTGT<br>GAAGCCCTGGACATAAGGGGCATGATGACTTGACGTCATCCCCACCTTCCTCCGAGTTG<br>ACCCCGGCAGTCCCCCACGAGTCCCCGACCGAATCGCTGGCAACATGGGGCAAGGGTT<br>GCGCTCGTTGCGGGACTTAACCCAACATCTCACGACACGAGCTGACGACAGCCATGCA<br>CCACCTGTACACCAACCACAAGGGAAACTATGTCTCCATAGCAATCTGGTGCATGTCAA<br>ACCCAGGTAAGGTTCTTCGCGTTGCATCGAATTAATCCACATGCTCCGCCGCTTGTGCGG<br>GCCCCCGTCAATTCCTTTGAGTTTTAGCCTTGCGGCCGTACTCCCCAGGCGGGGCGCTTA<br>ATGCGTTAGCTACGGCACGGACACCGTGGAACCAGTCCCCACACCTAGCGCCCAACGTT<br>TACGGCGTGGACTACCAGGGTATCTAATCCTGTTCGCTCCCCACGCTTTCGCTCCTCAGC<br>GTCAGTATCGGCCCAGAGACCCGCCTTCGCCACCGGTGTTCCTCCTGATATCTGCGCATT<br>TCACCGCTACACCAGGAATTCCAGTCTCCCCTACCGAACTCAAGTCTGCCCGTATCGAC<br>CGCAAGCCCACAGTTAAGCTGCAGGTTTTCACGGCCGACGCGACAAACCGCCTACGAG<br>CTCTTTACGCCCAATAAATCCGGACAACGCTCGCACCCTACGTATTACCGCGGCTGCTG<br>GCACGTAGTTAGCCGGTGCTTCTTCTACACCTACCGTCACCCGAAGGCTTCGTCGATGTC<br>GAAAGAGGTTTACAACCCGAAGGCCGTCATCCCCCACGCGGCGTTGCTGCGTCAGGCTT<br>TCGCCCATTGCGCAAGATTCCCCACTGCTGCCTCCCGTAGGAGTCTGGGCCGTGTCTCAG<br>TCCCAGTGTGGCCGGTCACCCTCTCAGGCCGGCTACCCGTCGTCGCCTTGGTAGGCCATC<br>ACCCCACCAACAAGCTGATAGGCCGCGGGCTCAACCTACACCGCCGGAACTTTCCACA<br>CACAGACCATGCGGCCATGCGTCCTATCCGGTATTAGACCCCGTTTCCAAGGCTTATCCC<br>AGAGTGCAGGGCAGATTACCCACGTGTTACTCACCCGTTCGCCACTCATCCACCCACCG<br>AAGCGGGCTTCAGCGTTCGACTGCAT

Table S2. The number of genes detected in each sample was counted

Sample Name	Known Gene Num
A22-37a	5580 (93.33%)
A22-37b	5537 (92.61%)
A22-37c	5565 (93.08%)
A22-42a	5569 (93.14%)
A22-42b	5661 (94.68%)
A22-42c	5563 (93.04%)
A22-50a	5474 (91.55%)
A22-50b	5542 (92.69%)
A22-50c	5503 (92.04%)

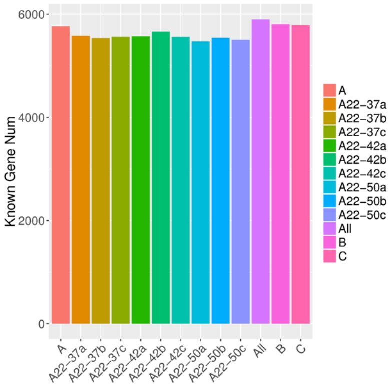

Figure S1. Statistical map of the number of genes identified.

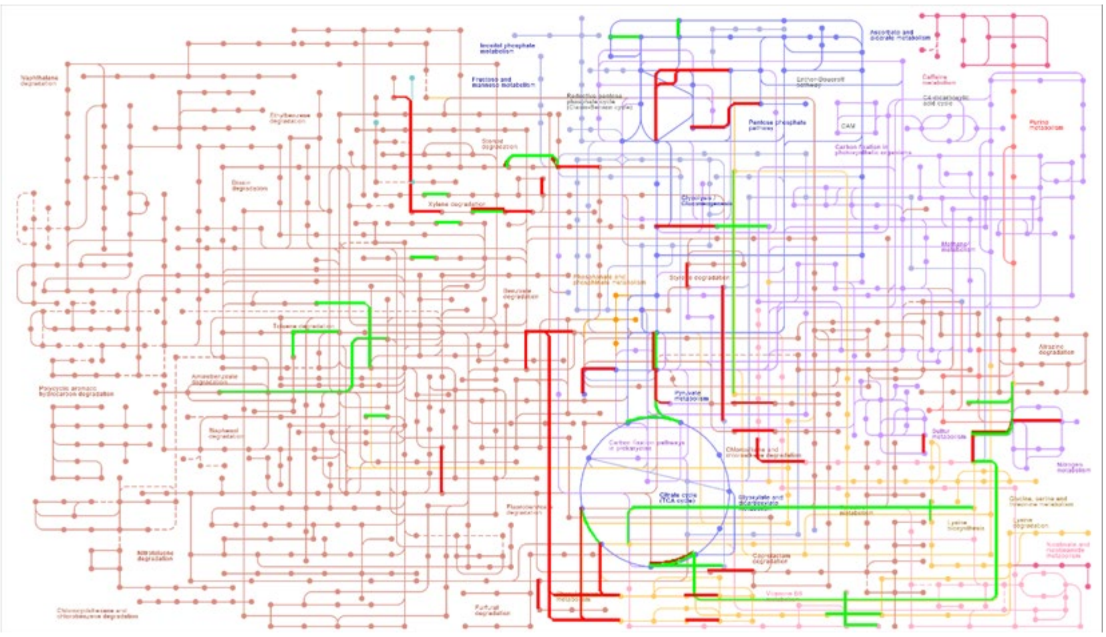

Figure S2. Microbial metabolism in diverse temperatures
